# Predictors of change in emotional regulation from 6 to 30 months of age in infants born after a threatened preterm labour.

**DOI:** 10.1192/j.eurpsy.2024.741

**Published:** 2024-08-27

**Authors:** J. Andreu, J. Buesa, L. Campos, B. Almansa, C. Zapata, M. Lizarán, N. Gómez, F. Ghosn, A. Moreno, A. García-Blanco

**Affiliations:** ^1^Psychiatry, Hospital Universitario y Politécnico La Fe de Valencia; ^2^Psychology, La Fe Research Institute; ^3^Psychology, Hospital Universitario y Politécnico La Fe de Valencia, Valencia, Spain

## Abstract

**Introduction:**

Emotional dysregulation are considered early manifestations of neuropsychiatric disorders. Recent research has shown that a threatened preterm labour (TPL) represents an adverse prenatal event that involves temperament disturbances, even in absence of prematurity. Thus, full-term TPL infants at 6 months of age are characterized by lower positive affect, higher negative affect, and worse emotional regulation relative to a full-term non-TPL control group.

**Objectives:**

The aim of this study is to explore the predictors of change of emotional infant competences.

**Methods:**

This prospective cohort study recruited mothers who suffered from a TPL. Infants’ temperament assessment was performed at 6 and 30 months of age using the Rothbart Behaviour Questionnaires, examining positive affectivity/surgency, negative emotionality, and orienting and emotional regulatory capacity. A regression model was carried out, including gestational age at birth, maternal anxiety trait, maternal history of psychological traumas, prenatal and postnatal maternal depression, anxiety, and cortisol as well as parenting stress as predictors.

**Results:**

Increased positive affectivity was related with lower paternal stress (p = .044). Maternal history of trauma and parenting stress was associated with increased negative emotionality (p = .037 and p = .045, respectively). Increased emotional regulation disturbance was linked to low gestational age at birth (p < .001), higher postnatal depression (p = .002), higher prenatal anxiety at TPL diagnosis (p = .039) and higher postnatal anxiety (p = .008).

**Conclusions:**

Therefore, maternal previous traumas, maternal psychopathology from pregnancy to postpartum as well as parenting stress should be considered in psychological treatment to improve infant’s emotional competences and prevent subsequent neuropsychiatric disorders.

**Disclosure of Interest:**

None Declared

